# From Blood to Regenerative Tissue: How Autologous Platelet-Rich Fibrin Can Be Combined with Other Materials to Ensure Controlled Drug and Growth Factor Release

**DOI:** 10.3390/ijms222111553

**Published:** 2021-10-26

**Authors:** Karina Egle, Ilze Salma, Arita Dubnika

**Affiliations:** 1 Rudolfs Cimdins Riga Biomaterials Innovations and Development Centre, Institute of General Chemical Engineering, Riga Technical University, LV-1658 Riga, Latvia; karina.egle@rtu.lv; 2 Baltic Biomaterials Centre of Excellence, Headquarters at Riga Technical University, LV-1658 Riga, Latvia; ilze.salma@rsu.lv; 3 Institute of Stomatology, Rīga Stradiņš University, LV-1007 Riga, Latvia

**Keywords:** platelet-rich fibrin, endogenous growth factors, carrier systems, drug delivery, platelet concentrates, tissue engineering, autologous growth factors

## Abstract

The purpose of this review is to examine the latest literature on the use of autologous platelet-rich fibrin as a drug and growth factor carrier system in maxillofacial surgery. Autologous platelet-rich fibrin (PRF) is a unique system that combines properties such as biocompatibility and biodegradability, in addition to containing growth factors and peptides that provide tissue regeneration. This opens up new horizons for the use of all beneficial ingredients in the blood sample for biomedical purposes. By itself, PRF has an unstable effect on osteogenesis: therefore, advanced approaches, including the combination of PRF with materials or drugs, are of great interest in clinics. The main advantage of drug delivery systems is that by controlling drug release, high drug concentrations locally and fewer side effects within other tissue can be achieved. This is especially important in tissues with limited blood supply, such as bone tissue compared to soft tissue. The ability of PRF to degrade naturally is considered an advantage for its use as a “warehouse” of controlled drug release systems. We are focusing on this concentrate, as it is easy to use in manipulations and can be delivered directly to the surgical site. The target audience for this review are researchers and medical doctors who are involved in the development and research of PRFs further studies. Likewise, surgeons who use PRF in their work to treat patients and who advice patients to take the medicine orally.

## 1. Introduction

Although many approaches to bone tissue engineering have traditionally focused on synthetic materials (such as polymers or hydrogels), nowadays new emerging methods involve the use of natural materials due to their biological properties, such as autologous bone grafting. It is still debated whether platelets can be considered as cell fragments or whole cells [1], but they are known to be responsible for the activation and release of biomolecules. The growth factors that accelerate the wound healing process. Due to this, the use of platelet concentrates has been known for more than four decades [2]. Platelet-rich fibrin (PRF) is an autologous material that is easily produced; it can be derived from a person’s own blood and is used to promote wound healing and tissue regeneration. PRF can be used in various fields of medicine, including dentistry and maxillofacial surgery [3]. PRF is expected to have a direct effect on enhancing tissue regeneration by saturating these tissues with growth factors from the blood. The autologous nature of PRF makes it the preferred choice for a variety of biomaterials in use today.

In this review, we considered 4 types of platelet concentrates as a possible drug delivery systems listed in Table 1.

Several studies have reported that these platelet concentrates are most commonly used in medicine. There are also articles on other platelet concentrates, such as pure platelet-rich plasma (P-PRP), leukocyte-platelet-rich plasma (L-PRP), pure platelet-rich fibrin (P-PRF), and leukocyte- and platelet-rich fibrin (L-PRF). These concentrates are used alone or in combination with bone grafts. To promote tissue regeneration the platelet concentrates should contain not only platelets, but also substances in our blood, such as growth factors and host immune cells. Various growth factors are known to promote wound healing, with PRF being able to release them slowly. In addition, when combined with drugs, they can provide faster recovery and reduce the risk of infections. Platelet-rich plasma contains large amounts of platelets and is injectable, while not as easy to prepare and use it [8].

The second type of platelets, platelet-rich fibrin (PRF), plays an important role in modern medicine and is used as one of the components in the production of biomaterials. As mentioned in several sources, PRF is a second-generation platelet concentrate derived from centrifuged blood [5] and, in addition to platelets, also contains white blood cells, serum and concentrated growth factors [9], such as platelet-derived growth factor (PDGF), transforming growth factor-β (TGF-β) and insulin-like growth factor 1 (IGF-I) [10]. The properties of growth factors and cytokines within the PRF are shown in Table 2.

PRF is widely used to accelerate soft and hard tissue regeneration [17]. This was first described by Choukroun and his group in 2001 in France [35]. The PRF production protocol originally developed by his group used 10 mL of anticoagulant-free blood sample that was centrifugated at 2700 rpm for 12 min [16]. PRF is a modification of platelet rich plasma (PRP) and at the same time an autologous fibrin matrix used to improve bone regeneration and clinically used for soft tissue augmentation [10]. Compared to other platelet concentrates, PRF is a platelet-rich fibrin clot that does not require the use of thrombin (anticoagulants which used to accelerate gelation), but only centrifuged blood without any impurities [36,37]. It is a new biomaterial that resembles an autologous cicatrical matrix, but at the same time is neither a fibrin glue or a classic platelet concentrate [38]. The absence of anticoagulants does not delay the cascade of wound healing allowing natural blood clots to form. In addition, PRF contains a high concentration of host immune cells, which are needed to heal wounds and reduce infections [39].

Compared to platelet-rich plasma (PRP) (it requires multi-stage centrifugation in combination with the addition of non-autologous anticoagulants and the additional use of bovine thrombin or calcium chloride [16]), PRF does not dissolve for following hours after application, on the contrary it is slowly destroyed in the same way as a natural blood clot [40]. As mentioned above, bovine thrombin or anticoagulant is not required to obtain PRF, thus PRF provides lower costs and fewer disadvantages of biochemical modifications [8]. After centrifugation, PRF still combines many of the healing and immune enhancers found in the initial blood [17]. After injection, unlike PRP, liquid PRF (i-PRF) is rapidly converted to fibrin and, similarly to PRP, i-PRF is used for the local delivery of autologous angiogenic and regenerative growth factors [2,41].

Different platelet-rich fibrin (PRF) derivatives are used today depending on the application and the desired properties. Efficacy of autologous platelet concentrates in promoting wound healing and tissue regeneration is at the center of a recent academic debate [42]. In this review, we will consider few of the PRFs mentioned above that have attracted the most attention as drug delivery systems, and will try to understand which type of PRF is better and more suitable for development of controlled drug delivery systems.

## 2. Materials and Methods

Articles were searched for keywords such as “platelet-rich fibrin”, “growth factors PRF”, “drug delivery systems PRF”, “platelet-rich fibrin”, “antibiotics PRF”, “drug PRF”, and “drug fibrin”. In case it was necessary to find other articles with the PRF that included the specified drug, then the name of the drug was used as a keyword. Emphasis on the literature related to PRF clinical trials and studies investigating drug incorporation, growth factor secretion was placed. Antibacterial studies to understand whether drugs can provide antibacterial efficacy by being included in the PRF matrices were also reviewed in relation to drug studies. Databases such as PubMed/MEDLINE, ScienceDirect, Scopus were used for search. In total, 200 studies were found for the above keywords, from which 121 articles were selected for further analysis in this review.

## 3. From Blood to Injectable or Solid System

**Figure 1:**
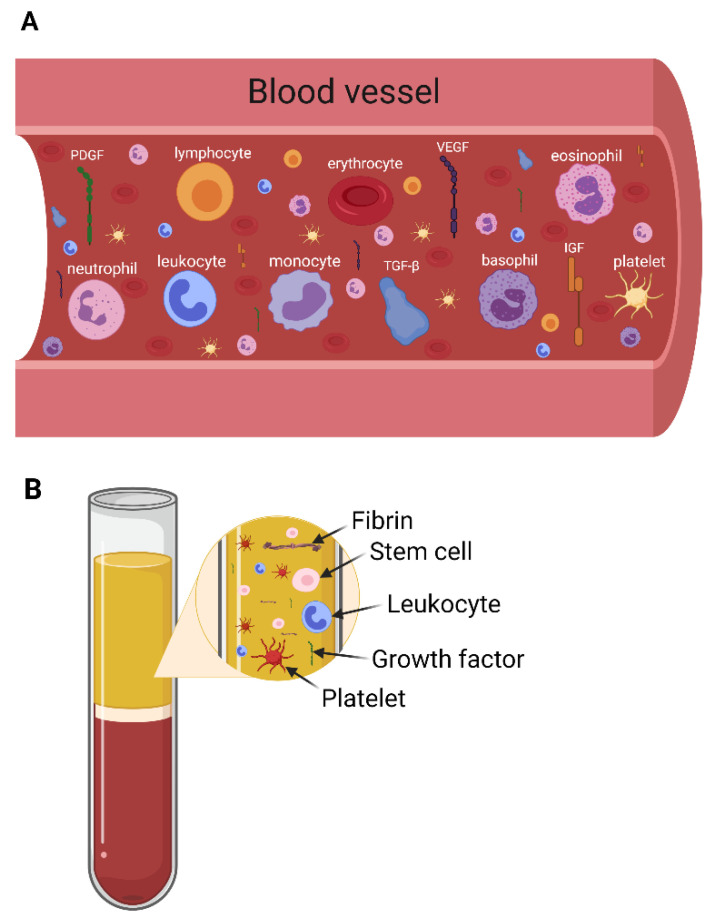
Main elements of blood (**A**) and PRF (**B**). Panel B shows that not all elements in the blood enter the PRF layer after centrifugation. Both figures created with Biorender.com.

i-PRF is liquid injectable PRF and allows the incorporation of drugs and drug delivery systems prior to coagulation. i-PRF is a recently introduced platelet concentrate [43] that can be easily combined with various biomaterials [44] to improve the properties of the biomaterial. i-PRF contains not only autologous growth factors found in the blood, but also cells involved in the wound healing process [45] (Figure 1). The functions of cells found in the PRF are shown in Table 3.

After about 20 min fibrin is polymerized (the liquid state of PRF depends on the speed, G-force and time of centrifugation), during which fibrin changes from a liquid state to a solid, forming a three-dimensional fibrin network. This network contains cellular components that are distributed in the network and ensure the slow and steady release of growth factors over a period of time. In addition, a controlled release system maintains bioactivity throughout healing [4]. As mentioned by Bennardo et. al., liquid PRF can also be used as an injection to treat lichens [58].

**Figure 2:**
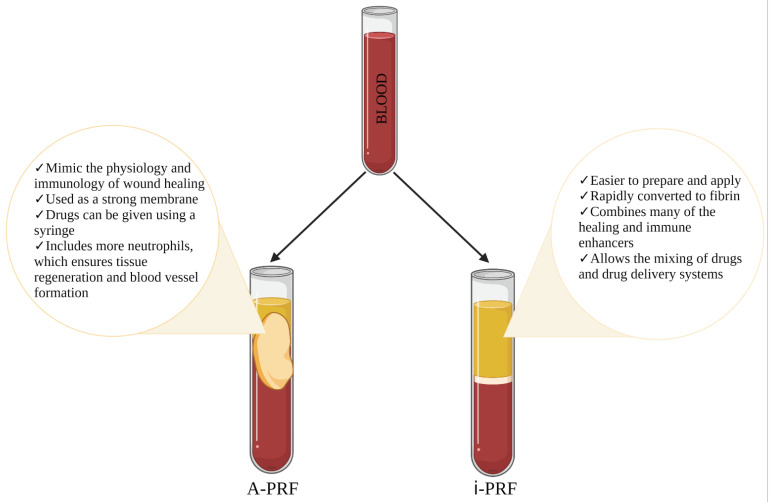
Comparison of the advantages for two concentrates i-PRF and A-PRF. Figure created with Biorender.com.

A-PRF, on the other hand, is a solid system (in the form of a clot) that can be compressed and used as a strong membrane. Modification of PRF preparation procedures at lower centrifugation rates with lower G-forces results in A-PRF with higher levels of growth factors [9]. This is indicated by a Caruana et.al group study on the variability of TGF-β1 and VEGF growth factor concentrations depending on the change in the PRF preparation protocol. At high relative centrifugal force (RCF), they obtained PRF with TGF-β1 less than 2000 pg/mL and VEGF equal to 0 pg/mL. The use of a moderate RCF protocol increases the concentration of growth factors, resulting in TGF-β1 greater than 2000 pg/mL and VEGF greater than 10 pg/mL. On the other hand, when RCF was reduced to low, a 2-fold increase in growth factors was observed, resulting in TGF-β1 > 4000 pg/mL and VEGF > 20 pg/mL [59]. Due to the high concentration of growth factors, A-PRF has the potential to mimic the physiology and immunology of wound healing [60]. During the production of A-PRF, it is important to maintain platelets, leukocytes, circulating stem cells and endothelial cells in the fibrin clot [9]. In the A-PRF obtained by the Choukroun et al. group method, the white blood cell count includes more neutrophils, which ensures tissue regeneration and vessel formation [61] due to its ability to promote the anti-inflammatory state of macrophages [60]. As mentioned by Wend et al., in addition to solid PRF, there is a clinical need to develop injectable PRF matrices for various clinical procedures and to improve angiogenic potential through the ability to combine i-PRF with various biomaterials [52]. Figure 2 shows the advantages of i-PRF and A-PRF. The idea of the review is to show that these 2 types of PRFs can be used as candidates for the development of drug delivery systems. That they are the ones that contain more growth factors that can ensure wound healing.

## 4. Therapeutic Enhancement of PRF

The most common postoperative risk of minor surgeries is infection caused by membrane exposure and colonization of wound bacteria [62,63]. PRF itself may show antibacterial activity, but it has not been relatively well studied and there are insufficient data on what affects it. The main unanswered questions are: 1. Does it depend on the concentration or on the characteristics of the patient’s blood? 2. If derived from a patient, then what properties are crucial to obtain a PRF antibacterial? There are also no data against which bacteria PRF itself may be antibacterial and which certain antibiotics must be added. In an attempt to delve into this issue, studies were found describing the antibacterial activity of L-PRF (leukocyte- and platelet-rich fibrin) [64,65] and H-PRF (PRF prepared by horizontal centrifugation) samples [65]. Another study looked at the antimicrobial properties of i-PRF against biofilm formation produced by certain *Staphylococcal* isolates, indicating the need to further investigate the antimicrobial properties of i-PRF based on an in vivo model [66]. This is also confirmed by other studies indicating that PRF has only mild antibacterial activity against some bacterial agents, including *S. aureus*, and does not show efficacy against resistant bacteria [67,68]. In turn, it is known that there are other bacterial isolates against which i-PRF would need to be antibacterial.

Oral administration of drugs is sometimes ineffective because absorption is irregular and incomplete in the most cases. Changes in drug solubility can occur as a result of reaction with other materials in the gastrointestinal tract. It is not suitable for emergencies where the medication must be taken as soon as possible, since the onset of action of the oral medication is relatively slow (long process from intake to destination) [69]. In addition, a significant advantage of local drug delivery is the ability to achieve high and stable local drug concentrations without high systemic doses, thereby reducing systemic toxicity [70]. Based on the collected literature on the antibacterial properties of PRF, we believe that it would be ideal to combine it with drugs to form a single system, rather than using separate drugs and PRF. In the last decade, there are few studies that combine antibiotics with PRF to provide an antibacterial effect.

The literature has reported that PRF is often combined with drugs, such as metronidazole, clindamycin, penicillin [71], vancomycin, teicoplanin, gentamicin, or amikacin to kill bacteria and speed up the healing process [72]. Today, in oral and maxillofacial surgery (including the prevention and treatment of osteonecrosis of the jaw [73]), there is an increasing demand for clindamycin as a drug. It is widely regarded as an alternative for patients who have an allergic reaction to penicillin [74]. Below we have shown the possible applications of PRF with drugs, the studies are systematized by drug classes.

The studies related to the combination of PRF with the drugs are shown in the Table 4.

### 4.1. Antibiotics

Antibiotics are considered to be an effective treatment for various types of infections caused by bacteria (gram-positive and gram-negative). In turn, their misuse can lead to antibiotic resistance [87]. Wound healing is a normal biological process in the human body, but in the postoperative period there is a high risk that there may be factors that will affect this process. It is important to ensure a proper healing process and reduce the risk of infections [88]. For wound healing, PRF can also be used as a drug carrier in another system. Therefore, we collected the literature in which PRF is used in combination with antibiotics to determine if PRF can provide antibiotic therapy at wound sites. One of the discovered studies described a method in which PRF together with one of 3 different drugs (amikacin, teicoplanin, and polyhexanide (a group of drugs used to treat wounds)) was sprayed onto a patch and tested for antimicrobial activity for 7 days. The results showed that for amikacin antimicrobial activity was observed up to 120 h, for teicoplanin —168 h, while for polyhexanide it can be observed only for 24 h [72]. This study indicates that it is possible to obtain a system in which certain drugs are dispensed at a specific time, providing the necessary therapy.

#### Lincosamides

This class of drugs is obtained from *Streptomyces spp.* [89]. Lincosamides are mainly used to treat anaerobic infections caused by gram-positive organisms, including infections developed by methicillin resistant *Staphylococcus aureus* [90]. Originally this class of antibiotics comes from natural product lincomycin, but derivatives also include clindamycin and pirlimycin, from which clindamycin is the most clinically relevant lincosamide [90,91]. Drugs such as lincomycin and clindamycin are bacteriostatic and inhibit protein synthesis. In the same way, both drugs are used in clinical practice and at higher concentrations in in vivo, they will become bactericide [92]. Clindamycin has been shown to be more effective than lincomycin in treating bacterial infections. In turn, doctors choose it for the treatment of odontogenic infections. The choice of doctors can be explained by the fact that clindamycin has not only bactericidal activity, but also significant tissue distribution and low resistance [93].

Summarizing the literature on current in vitro studies, it was found that there was one study in which lincosamide, a clindamycin, was included in the A-PRF. There is direct mixing of the drug in the blood sample and afterwards A-PRF clot was used. This system is relatively easy to obtain and can provide drug release for up to 4 days. However, in order to be used for further studies, it would have to be processed if a non-cylindrical sample was required. Additionally, this sample is not injectable, so it can be used as a ready-made clot, without the possibility to use to fill the defect site. In addition, this study lacks data on the effectiveness of drug encapsulation, which cannot predict what percentage of added drugs are encapsulated [71]. The effect of lincomycin form and volume on the antibacterial activity of PRF after 24 h, 48 h, 120 h, and 240 h was also studied using the drug in 3 forms (ampoule, capsule-mixed with saline and powder). The results showed that greater antibacterial activity was obtained by adding ampoule-type lincomycin to a blood sample [75,76].

### 4.2. Bisphosphonates

It is a group of medicines that are used in osteoporosis (a systemic skeletal disease that results in increased bone fragility and susceptibility to fractures [94]) and when the bone is not formed properly [95]. The most common condition that causes bone defects is periodontitis (an inflammatory disease of the supporting tissues of the teeth caused by certain microorganisms or groups of certain microorganisms [96]). It is believed that intrabony defects are less prevalent than horizontal bone loss. Intrabony defects pose a risk of disease progression and need to be treated. Looking at the literature, one gets the impression that not all intrabony defects can be cured. Due to the development of new biomaterials by scientists, it is likely that dental prognosis will be improved [97]. An interesting fact is that the combination of PRF with drugs can be used to treat this disease. Some studies [77,78] have described that PRF is used in combination with drugs, such as alendronate (ALN) to treat this disease. In the first study, combining 1% ALN gel with A-PRF, the researchers tried to prove the effectiveness of a combination of the two in treating grade II mandibular furcation defects compared to PRF. The PRF/1% ALN gel composition allowed to fill a higher percentage of defects (56.01 ± 2.64%), where simply for PRF therapy (49.43 ± 3.70%), indicating that the PRF/1% ALN combination has a recovery potential [77]. In the second study, the PRF/1% ALN combination is already being studied for the treatment of intrabony defect in chronic periodontitis. As in the previous study, the PRF/1% ALN combination was able to provide a greater reduction in defect depth (54.05 ± 2.88%) compared to PRF (46 ± 1.89%) [78]. As can be seen, both studies have similar results, confirming the reliability of the results of the PRF/1% ALN combination. This study indicates that the use of PRF in combination with a bisphosphonate drug can be used to reduce the size of the defect.

### 4.3. Statins

It is a class of drugs that is effectively used to lower cholesterol and also to reduce the risk of cardiovascular morbidity and mortality [98]. At the time of writing this report, studies were found where a representative of this class of drugs is used in the treatment of intrabony defects. Martande et al. in their study used 1.2% atorvastatin gel in combination with A-PRF and open flap debridement (OFD) to achieve the desired outcome [79]. Scientists have concluded that additional studies related to the use of PRF in the treatment of intrabony defects may reduce the number of patients with periodontitis. Another study also looked at the effect of the combination of the drugs and the PRF on clinical parameters. In this study alone, rosuvastatin (RSV) was selected as a drug, and the effects of open-flap debridement (OFD) with or without PRF and PRF/RSV were further investigated. In the present study, Pradeep at. al study results showed that combining RSV with PRF provided greater periodontal benefits compared to OFD alone or OFD/PRF [80]. The results of this study are similar to those of Pradeep et al. studies where RSV gel was used in combination with PRF and hydroxyapatite bone graft [99]. Both of the above studies indicate that statin drugs can be used not only for the treatment of blood and vascular diseases, but also for the treatment of other, no less common diseases, thus expanding the use of the drug.

### 4.4. Biguanides

Biguanides are a class of herbal drugs [100] classified as non-sulfonylureas that act directly against insulin resistance [101]. In addition to one of the drugs in the class of statins, biguanide drugs can be used to treat intrabony defects. As one of these drugs, Pradeep et al. used 1% metformin (MTF) in combination with A-PRF and OFD in their study [83]. As with statins, metformin has been shown to reduce periodontitis. A similar study was performed Taneja et.al, comparing the differences between PRF/MFT and PRF at 6 and 9 months [82]. A study was also found evaluating the possible use of OFD/PRF/MFT in the treatment of grade II mandibular furcation defects [81]. It has been shown that biguanide drugs in combination with PRF is widely used in periodontal therapy, which indicates the ability of PRF to be a drug delivery vehicle.

### 4.5. Non-Steroidal Anti-Inflammatory Drugs

This class of drugs is one of the most widely used therapeutic classes in clinical medicine [102]. Additionally, drugs in PRF can be incorporated not only by mixing in the blood or by mixing with i-PRF, they can be injected with a needle into the A-PRF clot obtained after centrifugation. This method was used by Pillai et al. in their study to administer diclofenac to test whether the PRF as a carrier would be able to deliver the drugs locally. Their study was more based on comparing pure PRF with PRF/diclofenac on the basis of clinical parameters (postoperative pain, swelling, soft tissue healing, and infection risk, etc.). The obtained results confirmed the hypothesis that PRF/diclofenac gel improves the clinical parameters, thus indicating that such a method of drug administration can improve the wound healing process and promote bone regeneration [84].

### 4.6. PRF Combination with Several Drugs

Another interesting fact is that PRF can combine not only one type of drugs, but also a mixture of several drugs. In the study where i-PRF was mixed with the triple drug mixture (metronidazole (MET), ciprofloxacin (CIP), minocycline (MINO)), release was observed for up to 28 days, but burst release was already observed within the first 24 h [85]. If a high concentration one of these drugs is included in the i-PRF with a view to prolonged therapy, it is likely that a toxic drug concentration will be released within the first 24 h and the required controlled drug therapy will not be achieved. The study also says that the simultaneous release of all three drugs takes only 14 days, followed by the release of only MINO and MET, resulting in the loss of i-PRF from its initial use.

### 4.7. PRF Combination with Materials and Drugs

Another way to restore diseased or damaged bones is bone grafting. Nowadays, it receives a lot of attention today due to the slow and difficult integration of the grafted material. Platelet concentrates are used to improve this integration process, which also accelerates bone and mucosal healing. PRF is no exception, which, regardless of the way it is used (membranes or fragments), can be used to protect a surgical site after surgery. To protect the bone graft from anaerobic bacterial infections, the Simonpieri team added metronidazole to the PRF membrane combinated with freeze-dried bone allograft. In addition, the combination of these two excipients improved the histological quality of bone tissue in the graft [86].

In most cases, the drugs are mixed into the PRF, creating the necessary material to treat a specific problem. In contrast, there is a study in which the drug is first taken orally, indicating that administration of the drug to a previously acquired PRF system is not the only way to improve antibacterial efficacy. This time, an attempt was made to determine whether L-PRF, prepared after a single dose of oral antibiotic, was able to produce significant antimicrobial activity within 48 h. After 48 h, no sterile area was observed, indicating that 1 dose of oral antibiotic was insufficient to provide 48 h of antimicrobial activity. The data suggest that most antibiotics are concentrated in plasma and that only a small proportion of them end up in the PRF [64]. The drug concentration in the PRF after an oral drug consumption should be determined. The calculated amount could be used as ground for further use of the drugs. Additionally, it has to be investigated, is the calculated amount of the drug is safe to use in medical practice.

Looking at all of the studies described above, there is a tendency to combine PRF with drugs. However, several of these studies show insufficient analysis and lack of data (drug release time and amount).

## 5. PRF as a Bioactive Agent in Different Matrices

**Figure 3:**
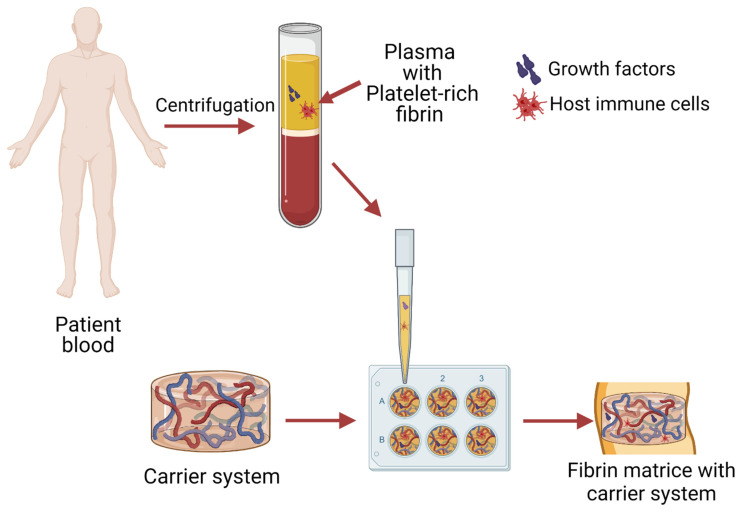
Principle scheme of platelet-rich fibrin as a carrier system preparation. Human blood is centrifuged by separating the PRF with a plasma layer. Obtained PRF is added it to pre-prepared carrier systems to obtain a PRF/drug carrier matrices. Figure created with Biorender.com.

One of the main requirements for carrier systems is the controlled release of the drugs and growth factors they contain (the bioactive molecule is delivered locally or systemically at a specific rate over a period of time). There are studies describing the successful combination of cells and growth factors or biomolecules with non-autologous fibrin. In turn, the autologous liquid i-PRF offers additional advantages as a carrier system for cells and growth factors [45] (Figure 3).

In this section, we have summarized the studies in which the PRF serves as a carrier system of bioactive molecules or was included in one of the carrier systems (Table 5).

PRF could not only serve as a carrier, but also be placed in another material. Xu et al. group described a study in which fresh granule-lyophilized platelet-rich fibrin (G-L-PRF) was incorporated into polyvinyl alcohol (PVA) hydrogels to improve wound healing. The results showed that increasing the G-L-PRF concentration could improve the mechanical strength and degradation rate of the scaffolds, but the concentration did not affect the flexibility and biocompatibility of the scaffolds. Regarding growth factors, the incorporation of G-L-PRF into PVA hydrogels provided a sustained and controlled release of growth factors from G-L-PRF/PVA scaffolds for up to 9 days [103]. Summarizing the literature, it was found that using PRF it is possible to develop a cell transplantation method. Such a method was developed in vitro by Zhao and his team using periodontal ligament stem cell (PDLSC) cell sheet fragments and PRF granules. The aim of this study was to improve periodontal healing in avulsed dental re-implantation. The results of the study showed that PRF induces significant and continuous stimulation of proliferation in human PDLSC throughout the 7-day incubation period, suggesting that the PDLSC/PRF construct may improve clinical outcomes in subsequent dental re-implantations. However, before using this method in patients, additional studies on the molecular mechanisms of the PDLSC/PRF interaction are required to ensure reliable results [104].

Returning to wound healing, growth factors are worth mentioning as they play an important role in the healing stages. Platelets are the main cell type in the inflammatory phase as they release PDGF and TGF-β. Both are growth factors that attract macrophages and neutrophils [113]. Therefore, it cannot be forgotten that PRF itself can serve as a matrices for the growth factors that are in every person’s blood. In their study, Ehrenfest et al. described three growth factors (TGFβ-1, PDGF-AB, VEGF) and coagulation matrix cellular protein, thrombospondin-1, (TSP-1), and the ability to release them in large amounts from the PRF membrane within 7 days. The results showed that comparing the amount of growth factors initially released to the end, it can be concluded that the leukocytes in PRF provide high TGFβ-1 and VEGF release throughout the experiment [105]. It turns out that PRF can be combined with other related materials to improve recovery. More specifically, this is described in a study by Yang et al., in which dental bud cells (DBC) were suspended in fibrin glue (used as one of the most effective scaffold materials) and then A-PRF was added. Thus, the restoration of dental tissue was achieved [106].

One of the studies is a double network (DN) hydrogel of i-PRF and gelatin nanoparticles (GHPs), with the aim to obtain a mechanically strong and bioactive hydrogel that can adapt to the irregular shape of the defect and withstand the required pressure. During this study, the release of growth factors (VEGF, platelet derived growth factor-BB (PDGF-BB), TGF-β and IGF-1) was observed for more than 3 weeks. This is higher compared to other studies [44,52] where in vitro release from pure i-PRF gel occurred in 2 weeks. In addition, DN hydrogels prevent the burst release of growth factors during the first hours [107]. The in vivo studies, described above, have shown that PRF matrices can be perceived as carrier systems due to their ability to release growth factors.

It should also be mentioned that the PRF can serve not only as a drug delivery system but also as a matrices of other materials. One ex vivo study analyzed the ability of the i-PRF matrix to be a autologous growth factor delivery system in combination with 5 collagen-based membranes. Thus, this was the first study that attempted to understand the ability and suitability of biomaterials to incorporate PRF. The assay was performed by separating leukocytes and platelets across the collagen membrane and determining the interaction between the collagen membrane and i-PRF. The obtained results showed differences in the structural composition of collagen membranes and differences in the interaction of collagen-based biomaterials with liquid PRF [108]. The obtained data confirmed the previous results that the interaction of the cell with the biomaterial is partially determined by the structural properties of the biomaterial [114,115]. Scientists have also tried to combine silk fibroin powder from *Bombyx mori* with Choukroun PRF. The results showed that the combination of these two materials can successfully prevent peri-implant defect [109]. Regarding the inclusion of other materials in PRF, a new approach to the use of PRF for the treatment of periodontitis defect has been explored. The approach is based on the placement of beta-tricalcium phosphate (β-TCP) granules at the furcation defect site, followed by the application of a PRF membrane covering both the defect site and the bone graft. Despite the successful results, this method requires additional research to ensure its suitability [110]. While searching for articles on the treatment of periodontitis, we found that PRF in combination with other materials can also be used to treat intrabony defects. In one such study, researchers combined PRF with an anorganic bovine bone (material for transplantation into alveolar cavities after human extraction [116]) mineral (ABBM), indicating that it is effective in treating these defects and may increase the rate of clinical attachment [111]. A similar study was carried out by the Lekovic group, where A-PRF was combined with bovine porous bone mineral (BPBM) instead of ABBM. Combining BPBM with A-PRF resulted in significantly greater reductions in pocket depth, increased clinical attachment, and defect filling than PRF used alone [112].

Summarizing all the above studies, it is observed that when using PRF as a matrices or including it in another carrier system, there is no need to add growth factors, as PRF itself includes certain growth factors. The only thing to consider, then, is the encapsulation of the desired drug and its interaction with other carriers that will be included in the PRF. It is also important to investigate whether the used carrier system will be able to ensure the controlled release of the growth factors that are in the PRF.

## 6. Conclusions and Future Perspectives

Summarizing the literature on the possible application of PRF, it has been observed that nowadays there is a growing demand for its application in operations. Several pieces of clinical research shows that PRF can be used in different surgeries, such as open-heart surgery, cranial surgery, endodontic surgeries, and periodontitis [117]. This allows surgeons to use the beneficial properties of PRF to solve a given problem, such as closing a defect and improving recovery. PRF is also widely studied as a drug delivery system to reduce the risk of postoperative infections.

Although platelet-rich fibrin is autologous and contains growth factors and cells, its antibacterial properties are not specifically expressed. In addition, analgesics, anticancer, and other therapies that would otherwise be administered intravenously or orally may be added to the PRF. For optimal drug use, it is necessary to study the effect of interaction between PRF and drug on controlled release of the drug and the ability of the sample to retain properties, such as biocompatibility, biodegradability, mechanical strength, and shape retention. Already additional biomaterials are being added to the PRF to provide these properties. However, there is a need to further explore the ability of this biomaterial to be a drug delivery system, combining the ability of PRF to retain growth factors and incorporate drugs.

Current research shows that most drug or drug delivery systems are mixed with the A-PRF clot or its membrane, and the amount of growth factors or the antibacterial activity of the material is studied. It seems that studies of the kinetics of drug release from the investigated samples are insufficient. Therefore, we propose to continue the study of i-PRF as a matrix for drug delivery systems, including liquid i-PRF before coagulation, and to test the ability of the material to provide controlled drug delivery. Only an understanding of the ability of these materials to be combined with other biomaterials and drugs will allow us to obtain new biomaterials with the necessary properties for use not only in maxillofacial surgery, but also in healing burns, neurosurgery, cartilage and tendon repair, and other fields.

## Figures and Tables

**Table 1:** Abbreviation of different platelet concentrates.

Abbreviation	Platelet Concentrate	Explanation
PRP	Platelet-rich plasma	First-generation platelet concentrate with high platelet concentrations [4]
PRF	Platelet-rich fibrin	Second-generation platelet concentrate [5]
i-PRF	Injectable platelet-rich fibrin	Advanced version of PRF in liquid form which can be injected and contains stem cells with high regenerative potential [6]
A-PRF	Advanced platelet-rich fibrin	An autogenous blood product with applications in dento-alveolar surgery [7]

**Table 2:** Description of growth factors and cytokines within the PRF.

Abbreviation	Growth Factor/Cytokine	Properties
PDGF	Platelet-derived growth factor	Provides fibroblast chemotaxis [11], extracellular matrix modification [12], and increases TGF-β release from macrophages [13]. Its addition ensures the growth of cultured cells [14] and improves bone cell proliferation [15]
TGF-β	Transforming growth factor β	A multifunctional cytokine [16] and one of 30 members of the superfamily [5] that has been shown to promote extracellular matrix formation [15]. The most common of the three isoforms [13] of TGF-β is TGF-β1, which has the ability to stimulate the production of collagen and fibronectin in cells [17]
IGF-I	Insulin-like growth factor I	A growth hormone-dependent polypeptide that stimulates skeletal growth in vivo [18], has an effect on the behavior of cells, thus providing tissue regeneration [19]
VEGF	Vascular endothelial growth factor	Promotes the proliferation [20] of endothelial cells and stimulates their migration [21]. It plays an important role in the cardiovascular system, increasing blood flow and enriching the injury site with nutrients [22]. In addition, it plays a role in bone formation and wound healing [23]
IL-1β	Interleukin-1β	Plays an important role in protection against infections and injuries [24], it is also involved in the activation of monocytes [25]
IL-6	Interleukin-6	Able to respond to infections and tissue injuries by stimulating hematopoiesis [26]. The main signal enhancement pathway [20] upon exposure to epithelium and immune cells [27]
IL-4	Interleukin-4	Acts as a powerful immune regulator [28] that inhibits the proliferation of osteoblast-like cells in vitro [29] and modulates the regeneration of macrophage cells [30]. It is also able to stimulate the accumulation of extracellular matrix macromolecules [31]
TNF-α	Tumor necrosis factor-α	Provides growth and differentiation of different cell types [32]. Stimulates the ability of fibroblasts to transform [20], and regulates the activity of vascular endothelial cells and keratinocytes. Determines the synthesis of extracellular matrix proteins [33]; it plays a key role in healing inflammation and wounds [34]

**Table 3:** Description of cells within the PRF.

Cell Type	Functions
Platelets	Involved in primary wound closure and able to release several growth factors to attract inflammatory cells to the site of injury [46,47]
Leukocytes	Essential for tissue regeneration as they direct and attract different types of cells in the wound healing process [44]
Red blood cells	Physical and chemical interactions between platelets and the blood surface may be provided [48]. Induces an increase in platelet concentrations at the site of action and in vitro coordination [49]
Neutrophils	Play an important role in healing processes [50]. Serves as the first signals for the activation of local fibroblasts and keratinocytes [51]
Lymphocytes	It affects the osteogenic differentiation of mesenchymal stromal cells [52] and releases a wide range of cytokines [53]
Monocytes	A key role in supporting tissue homeostasis by disseminating immune responses to convenience [54]
Stem cells	Play an important role in regenerative medicines [55], also have the opportunity to regenerate and differentiate in different types of cells [56]. PRF is a unique source of hematopoietic stem cells (HSCs) [57]

**Table 4:** Various drugs for inclusion in platelet-rich fibrin system (summary of the drugs in platelet-rich fibrin system).

Drug	Incorporation Method	Time of the Study	Reference
Clindamycin	Drug mixing in a blood sample, use of PRF clot	4 days	[71]
Lincomycin	Drug mixing in a blood sample, use of PRF clot	10 days antibacterial activity	[75,76]
Amikacin, teicoplanin or polyhexanide	PRF mixing with drug, using co-delivery applicator	168 h for amikacin, 120 h for teicoplanin and 24 h for polyhexanide antimicrobial effect	[72]
1% Alendronate gel	PRF combinated with drugs	9 months	[77,78]
1.2% Atorvastatin	Drug combination with PRF and open flap debridement (OFD)	9 months	[79]
1.2% Rosuvastatin gel	Drug gel adding into PRF membrane	9 months	[80]
1% Metformin	Drug combination with PRF and OFD	9 months	[81,82,83]
Diclofenac sodium	Drugs injected in PRF using needle	7 days	[84]
Triple antibiotic mixture (MET + CIP + MINO)	Antibiotic mixture mixing with i-PRF, i-PRF scaffold prepare	28 days	[85]
0.5% Metronidazole	Metronidazole added to the PRF membrane combinated with freeze-dried bone allograft	10 weeks	[86]
Amoxicillin	Drugs used orally 1 h before blood collection	48 h	[64]

**Table 5:** Carrier systems incorporated in injectable platelet-rich fibrin.

Carrier System	Target	Incorporation Method	Time of the Study	Reference
G-L-PRF	Accelerate wound healing	Fresh lyophilized PRF added to PVA hydrogels (simple physical method)	9 days	[103]
PRF granules	Improve periodontal healing	PDLSC cultivated with PRF membrane	7 days	[104]
PRF membrane	Improve wound healing	TGFβ-1, PDGF-AB, VEGF and TSP-1 included in PRF	7 days	[105]
Fibrin glue	Enrich the microenvironment with growth factors	Adding PRF into DBC/fibrin glue	36 weeks	[106]
Gelatin nanoparticles	Get mechanically tough and bioactive hydrogel	Mixing i-PRF with GNPs by repetitive extrusion	3 weeks	[107]
Collagen membrane	Enhance the bioactivity of collagen-based biomaterials	Liquid-PRF is applied to collagen membrane	24 h	[108]
PRF	Prevent peri-implant defect	Silk fibroin mixing with PRF in vivo	8 weeks	[109]
PRF membrane	Treatment of furcation defect	β-TCP granules insertion at the defect site and sealing with a PRF membrane	9 months	[110]
PRF membrane	Treatment of intrabony defects	ABBM mixed with PRF	6 months	[111]
PRF membrane	Treatment for periodontal intrabony defects	BPBM mixed with PRF	6 months	[112]

## Data Availability

No applicable.
